# Multi‐criteria manufacturability indices for ranking high‐concentration monoclonal antibody formulations

**DOI:** 10.1002/bit.26329

**Published:** 2017-05-26

**Authors:** Yang Yang, Ajoy Velayudhan, Nina F. Thornhill, Suzanne S. Farid

**Affiliations:** ^1^ Centre for Process Systems Engineering, Department of Chemical Engineering Imperial College London, South Kensington Campus London SW7 2AZ UK; ^2^ The Advanced Centre for Biochemical Engineering, Department of Biochemical Engineering University College London, Torrington Place London WC1E 7JE UK

**Keywords:** data mining, high‐concentration mAb formulation, manufacturability index, viscosity, aggregation, developability assessment

## Abstract

The need for high‐concentration formulations for subcutaneous delivery of therapeutic monoclonal antibodies (mAbs) can present manufacturability challenges for the final ultrafiltration/diafiltration (UF/DF) step. Viscosity levels and the propensity to aggregate are key considerations for high‐concentration formulations. This work presents novel frameworks for deriving a set of manufacturability indices related to viscosity and thermostability to rank high‐concentration mAb formulation conditions in terms of their ease of manufacture. This is illustrated by analyzing published high‐throughput biophysical screening data that explores the influence of different formulation conditions (pH, ions, and excipients) on the solution viscosity and product thermostability. A decision tree classification method, CART (Classification and Regression Tree) is used to identify the critical formulation conditions that influence the viscosity and thermostability. In this work, three different multi‐criteria data analysis frameworks were investigated to derive manufacturability indices from analysis of the stress maps and the process conditions experienced in the final UF/DF step. Polynomial regression techniques were used to transform the experimental data into a set of stress maps that show viscosity and thermostability as functions of the formulation conditions. A mathematical filtrate flux model was used to capture the time profiles of protein concentration and flux decay behavior during UF/DF. Multi‐criteria decision‐making analysis was used to identify the optimal formulation conditions that minimize the potential for both viscosity and aggregation issues during UF/DF. Biotechnol. Bioeng. 2017;114: 2043–2056. © 2017 The Authors. *Biotechnology and Bioengineering* Published by Wiley Perodicals, Inc.

## Introduction

The dominance of monoclonal antibodies (mAbs) in biopharmaceutical pipelines combined with their success in treating chronic conditions has triggered a shift in their final dosage form and delivery mode to high‐concentration formulations. Typical formulation studies explore the responses of the final product to different formulation conditions but do not typically translate these to predicting the ease of manufacture. Yet this becomes important to understand especially with high‐concentration formulations that have the potential to pose manufacturing challenges. Hence this paper proposes a novel methodology to predict manufacturability in the final ultrafiltration/diafiltration (UF/DF) step of a mAb process based on biophysical data derived from formulation studies.

Traditionally mAbs have been formulated at low concentrations (e.g., 20 g/L) for intravenous administration in hospitals (see Table I). More recently, high‐concentration and low‐volume (<1.5 ml) formulations have been developed (e.g., 100 g/L) for sub‐cutaneous delivery that can be self‐administered in the home and thus lower hospital administration costs while enhancing the ease of administration and hence patient compliance (Harris et al., 2004; Rao and Gefroh, 2012). Table I provides a list of commercial mAb‐based products with their concentrations and routes of administration.

**Table I:** Commercial antibodies with concentrations and routes of administration Adapted from Meyer and Shameem (2012)

Name	Trade Name	Company	Active concentration (g/L)	Dose per container (mg)	Mode of administration
Adalimumab	Humira	Abbott Labs (North Chicago, IL)	50	20, 40	SC
Basiliximab	Simulect	Novartis (Basel, Switzerland)	4	10	IV
Bevacizumab	Avastin	Genentech (South San Francisco, CA)	25	100, 400	IV
Canakinumab	Ilaris	Novartis (Basel, Switzerland)	150	150	SC
Certolizumab	Cimzia	UCB (Brussels, Belgium)	200	100	SC
Pegol					
Cetuximab	Erbitux	Eli Lilly and Co (Indianapolis, IN)	2	100, 200	IV
Denileukin diftitox	Ontak	Ligand Pharma (San Diego, CA)	0.15	0.3	IV
Denosumab	Prolia	Amgen (Thousand Oaks, CA)	60	60	SC
Golimumab	Simponi	Janssen Biotech (Horsham, PA)	100	50	SC
Ibritumomab	Zevalin	Spectrum Pharmaceuticals (Henderson, NV)	1.6	3.2	IV
Infliximab	Remicade	Janssen Biotech (Horsham, PA)	10	100	IV
Muromomab	Okt3	Janssen Biotech (Horsham, PA)	1	5	IV
Ofatumumab	Arzerra	GlaxoSmithKline (Seattle, WA)	20	100	IV
Omalizumab	Xolair	Genentech (South San Francisco, CA)	125	202.5	SC
Palivizumab	Synagis	MedImmune (Gaithersburg, MD)	100	50, 100	IM
Panitumumab	Vectibix	Amgen (Thousand Oaks, CA)	20	100, 200, 400	IV
Ranibizumab	Lucentis	Genentech (South San Francisco, CA)	10	2	II
Rinolacept	Arcalyst	Regeneron (Eastview, NY)	80	220	SC
Tocilizumab	Actemra	Genentech (South San Francisco, CA)	20	80, 200, 400	IV
Tositumomab	Bexxar	GlaxoSmithKline (Seattle,WA)	14	35, 225	IV
Trastuzumab	Herceptin	Genentech (South San Francisco, CA)	21	440	IV
Ustekinumab	Stelara	Janssen Biotech (Horsham, PA)	90	45, 90	SC

SC for subcutaneous injection; IV for intravenous infusion; II for intravitreal injection; IM for intramuscular injection.

High‐concentration formulations of mAbs can pose manufacturing and delivery challenges. These can be attributed to the higher propensity of antibodies to form soluble aggregates and to be more viscous at high concentrations given the higher probability for protein‐protein interactions (Arakawa and Timasheff, 1985; Shire, 2009; Shire et al., 2004; Treuheit et al., 2002). For example, it has been reported that viscosity values above 10 cP were observed with mAbs at concentrations above 100–150 mg mL^−1^ (He et al., 2010a; Monkos and Turczynski, 1999; Patapoff and Esue, 2009).

On the manufacturing front, biopharmaceutical manufacturers (He et al., 2011c; Rao and Gefroh, 2012; Shire, 2009) have commented that high concentration formulations present downstream processing challenges, in particular for the final ultrafiltration/diafiltration stage that uses tangential flow filtration (TFF) to buffer exchange and concentrate the product to meet the final product specification. Moreover, Shire et al. (2004) and Rao and Gefroh (2012) observed that the higher viscosities at higher concentrations may make recovery of the final product from the UF/DF step more difficult and hence result in excessive product losses. It may also result in high back‐pressures during the UF/DF process that can affect the system pumps and reduce the flux (Shire et al., 2004). Therefore, selection of formulation conditions and excipients that reduce the viscosity and minimize the propensity to aggregate is not only a priority for formulation studies considering storage and delivery of high concentration products but also for attaining feasible manufacturing processes given equipment limitations. Hence a closer linkage between formulation studies and manufacturability is needed to successfully realize high concentration formulations.

Formulation studies typically examine the impact of stress conditions such as pH, temperature, concentration, ions, and excipients on protein properties such as viscosity and stability. Reported strategies to reduce viscosity include control of pH in relation to the pI (Webb et al., 2002; Winters et al., 1996; Yadav et al., 2010a) and the addition of excipients including cations and anions to increase the ionic strength of the formulation (Kanai et al., 2008). However, designing an appropriate formulation can be complicated since some formulation strategies to decrease viscosity can also increase aggregation by lowering the thermostability of the protein solution (He et al., 2010b). Therefore, the optimal strategy design would aim to select formulation conditions that minimize viscosity while maximizing thermostability. A key challenge is considering how to map the stress responses obtained in formulation studies onto the manufacturing process. This will enable selection of formulation strategies that can also help avoid manufacturing complications when handling high‐protein concentrations.

This paper presents a novel concept of manufacturability indices that can be used as predictors in early stage development to rank high‐concentration mAb formulation conditions in terms of their ease of manufacture and formulation with respect to minimizing viscosity and aggregation issues during the final UF/DF step. This work builds on the notion of manufacturability indices first proposed by the authors in Yang et al. (2015) and presents multiple advanced frameworks for predicting the ease of manufacture. In the present work, published high‐throughput biophysical screening data that explored the influence of different formulation conditions (pH, ions, and excipients) on the solution viscosity and product thermostability were used for the study. The CART decision tree classification method was applied to identify the major formulation conditions influencing viscosity and thermostability. A polynomial regression method was applied to transform the experimental data into a set of stress maps which indicate viscosity and thermostability as functions of the formulation conditions. A mathematical model from Ho and Zydney (2000) for the expected flux decay behavior during membrane filtration was adapted and incorporated to capture the impact of protein concentration‐time profiles on manufacturability. The stress maps are thereby transformed into functions of the formulation conditions and processing time during UF/DF. Three different frameworks for generating manufacturability indices are demonstrated in order to address different user priorities. The three frameworks centered on derivation of either quantified overlay regions or temporal operating windows or temporal multi‐criteria weighted scores. The work demonstrates that these frameworks provide a systematic and holistic approach to leverage formulation screening data so as to predict manufacturability. It is envisaged that advances in high‐throughput assays and automation may allow such formulation studies to be carried out earlier in the development cycle, potentially across multiple candidates in the drug discovery phase. Hence the approaches presented in this work have also potential utility to be incorporated not only into early formulation studies but also into molecular/developability assessment exercises so as to de‐risk technical development.

This paper is organized as follows: first, the methods applied in the case study including CART decision tree and regression analysis are briefly introduced. Second, the published experimental DoE data used in the case study is summarized and the practical problems to be solved by the case study are described. In the Section Decision Tree Analysis Results, the key factors impacting viscosity and thermostability are derived using CART decision trees. In the Section Manufacturability Index Results, three proposed methodologies are analysed for the derivation of manufacturability indices. These indices consider the impact of the protein concentration‐time profiles on manufacturability, whilst accounting for the expected flux decay behavior during UF/DF. Multivariate regression and calculus techniques are used to transform experimental data into a set of viscosity and thermostability stress maps as a function of the formulation conditions and the time profiles during UF/DF and finally to derive the indices.

## Methodology

### Classification and Regression Tree (CART)

Classification and regression tree (CART) is a nonparametric procedure to construct prediction models and to establish splitting rules (Breiman et al., 1984; Grajski et al., 1986). CART divides the data into homogenous subsets using binary recursive partitions. The most discriminative variable is first selected as the root node to partition the data set into branch nodes. The root nodes and branch nodes in this study represent critical formulation parameters affecting protein solution viscosity and thermostability. The partitioning is repeated until the nodes are homogenous enough to be terminal nodes, which are called leaves. The terminal nodes represent critical ranges for the output metric of interest. So in a tree structure, each leaf represents a class label and each branch represents the splitting rules that lead to those class labels.

CART is able to convert large complex datasets into easy‐to‐understand and yet information‐rich graphical displays with minimal requirements for data preparation and robust performance on large datasets. Furthermore, CART has been demonstrated for biomanufacturing facility fit analysis to explore the impact of these process fluctuations on product mass loss and reveal the root causes of bioprocess bottlenecks (Yang et al., 2014). In the current work, CART was introduced to identify the major factors affecting viscosity and thermostability.

### Regression Analysis

Regression analysis is a statistical modeling approach for estimating the relationships among variables and is widely used for prediction and forecasting. Regression attempts to model the relationship between a set of input variables (independent variables) and a set of output variables (dependent variables).

In this work, polynomial regression has been used to analyze the DoE data and derive polynomial equations describing the relationship between viscosity and formulation factors. These polynomial equations have been used for manufacturability index calculations at a later stage. Furthermore, according to the impact factor analysis in the literature (He et al., 2011c), polynomial models can fit the DoE data well.

## Case Study Description

### Formulation DoE Datasets

The datasets used in this work have been published by He et al. (2011c). The datasets describe the high‐throughput measurements of thermostability and viscosity for an IgG2 mAb sample assessed using differential scanning fluorimetry (DSF) and dynamic light scattering (DLS) methods respectively (Shukla et al., 2007). He et al. (2011c) obtained the data via a full factorial design of experiments (DoE) with four predictor variables, including two continuous variables (pH and formulation concentration) and two categorical variables (presence of ions and excipient). Table II describes the summary of DoE predictor variables.

**Table II:** Summary of DoE predictor variables

DoE predictors	Variables		
pH	5.0	5.5	6.0
Formulation concentration (g/L)	89	119	149
Ion	N/A	Ca^2+^	Mg^2+^
Excipient	N/A	Sucrose	Proline

CaCl_2_ and MgCl_2_ were included at 50 mM. Final concentration of sucrose or proline was 200 mM.

### Problem Domain

For high concentration mAb manufacture, the key challenge is the final UF/DF step due to high viscosity. A typical mAb concentration entering the final UF/DF step is 5–10 g/L (Pollock et al., 2013). However, at the final UF/DF step, the mAb needs to be concentrated to the final desired concentrations which usually exceeds 100 g/L (Harris et al., 2004). Furthermore, in order to reduce the mass loss caused by hold‐up volumes (e.g., in piping) of TFF skids, Rao and Gefroh (2012) reported that some manufacturers have introduced extra overconcentration and flush sub‐steps in the final UF/DF step so as to recover as much as possible of the product held outside of the retentate tank in large‐scale skids. That means in high concentration protein manufacturing processes, the product concentration during the final UF/DF step is even higher than the target concentration. This poses a challenge because of the high viscosity and propensity to aggregate at the higher concentrations. Consequently, the product viscosity and thermostability levels need to be within acceptable limits beyond the target product concentration to be able to cope with higher stresses experienced during the UF/DF step. Hence it is important to be able to identify the optimal formulation conditions that reduce the solution viscosity and enhance the product thermostability during the final UF/DF step so as to avoid manufacturing obstacles.

In this case study, the required final product concentration is 100 g/L. The concentration after entering the UF/DF step is 5 g/L. The overconcentration ratio in the final UF/DF step is 1.5 which means the product concentration is 150 g/L after the overconcentration step. The influence of different formulation conditions on solution viscosity and thermostability has been explored by high‐throughput DoE experiment as described in the Section Formulation DoE Datasets. The aim of this work is to rank different formulation designs according to their potential UF/DF manufacturability and to choose the optimal formulation conditions that minimize the potential for viscosity issues while meeting the thermostability requirement.

**Figure 1:**
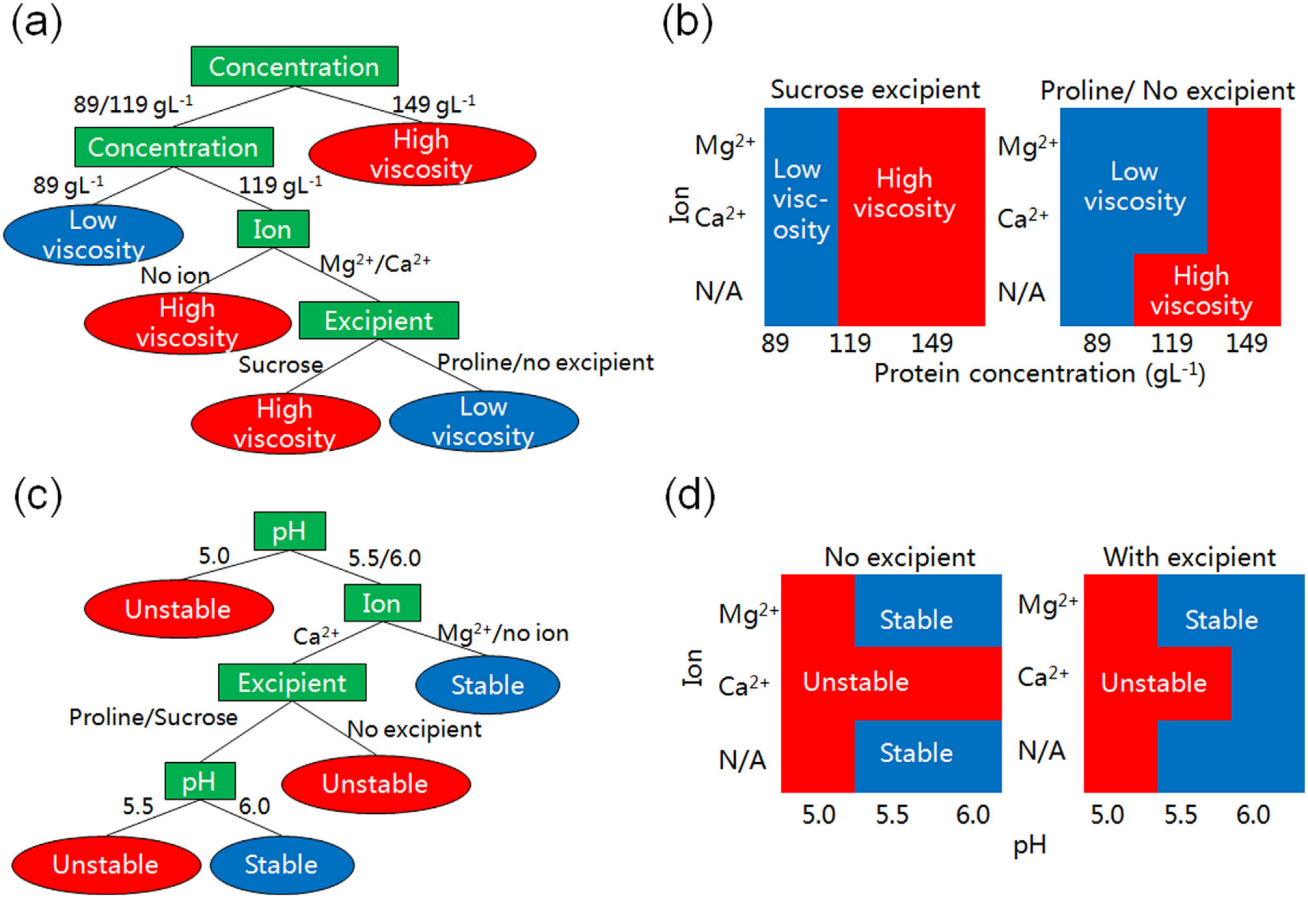
Data mining results for formulation conditions driving viscosity and thermostability. **a**) CART tree for viscosity factors analysis. Rectangular nodes are branch nodes (e.g., concentration, pH) which represent the formulation factors leading to split. Values on branches are the threshold levels of the split points for the corresponding split conditions. Circular nodes are leaves representing subsets with different class labels for viscosity (solution viscosity is higher or not than 6 cP). **b**) Impact of different combinations of formulation factors on viscosity with sucrose excipient or proline/no excipient. The blue area represents formulations with low solution viscosity (<6 cP), and the red area represents formulations with high solution viscosity (≥6 cP). **c**) CART tree for thermostability analysis. Circular nodes are leaves representing subsets with different class labels for thermostability (*T*_h_ is higher or not than 50 °C). **b**) Impact of different combinations of formulation factors on thermostability, with no excipient and with excipient (sucrose or proline excipient). The red area represents formulations unstable at 50 ^°^C (*T*_h_ < 50 °C), blue area represents formulations stable at 50 °C (*T*_h_ ≥ 50 °C).

## Decision Tree Analysis Results

### CART Tree for Identification of Key Factors Influencing Viscosity

As described in Section Formulation DoE Datasets, the published viscosity DoE dataset has 80 data records. Each data record represents one formulation design with a combination of four variables: pH, formulation concentration, presence of ions, and excipient. Each data record in the DoE dataset was assigned a class label of either high viscosity (≥6 cP) or low viscosity (<6 cP). This enabled the supervisory learning method used by the decision tree classification to identify key contributory factors leading to each outcome. The threshold value used for high viscosity was the set to be the same as that used in the original data source (He et al., 2011c). Although the critical viscosity value (6 cP) may be considered low, recent studies have demonstrated that the viscosity of mAb solutions can experience sharp exponential increases with mAb concentrations, particularly above 100 g/L and above viscosities of 5–10 cP (Yadav et al., 2010b). Hence the threshold value used in the analysis could be considered industrially relevant but should not be seen as definitive. It was selected with the primary aim of demonstrating the application of the proposed methodologies to transform formulation datasets into predictive manufacturability assessments and enable comparisons with previous work using the same data source (He et al., 2011c).

In Figure 1(a), the CART tree for the viscosity dataset reveals that the most critical factors influencing the solution viscosity are formulation concentration followed by presence of ions and finally presence of excipients. The pH values have less impact on viscosity in this particular dataset. In order to clearly display the relationship between the viscosity distribution and key factors based on the decision tree prediction model, plots of formulation concentration versus presence of ions under the presence of different excipients were generated in Figure 1(b). Several conclusions can be drawn from examination of Figures 1(a) and (b):

- Viscosity is highly related to formulation concentration rather than other factors. When the formulation concentration is low (89 g/L), low viscosity (<6 cP) is expected irrespective of the other factors. On the other hand, when the formulation concentration is high (149 g/L), viscosity will be high (≥6 cP) irrespective of the other factors. This observation reinforces literature reports that protein solution viscosity increases rapidly with increasing concentration due to the reversible self‐association and weak intermolecular interactions between protein molecules at high concentration (Liu et al., 2005, Yadav et al., 2010a, Yadav et al., 2010b).
- For a medium formulation concentration of 119 g/L, the presence of Ca^2+^ and Mg^2+^ ions can reduce viscosity to lower than 6 cP in the absence of sucrose excipient. The impact of ions can be explained by the interaction between charged ions and charged amino acid side chains on the protein surface which alters protein–protein interactions and decreases the solution viscosity (He et al., 2010a; Kanai et al., 2008; Liu et al., 2005).
- Compared to proline, the presence of sucrose excipient can lead to higher viscosity especially at the medium formulation concentration of 119 g/L. This effect of sucrose on the solution viscosity has been reported in previous work and has been explained by the interactions between sugar and protein molecules in solution (He et al., 2011b; Kanai et al., 2008).

### CART Tree for Identification of Key Factors Influencing Thermostability

Similar to the viscosity dataset, according to the values of hydrophobic exposure temperature (*T*_h_), the thermostability dataset with 80 data records was classified into one of two groups: stable (≥50 °C) and unstable (<50 °C). The low limit of hydrophobic exposure temperature was set at 50 °C according to the literature source (He et al., 2011c). The CART tree is shown in Figure 1(c). The tree shows that pH followed by presence of ions and then presence of excipients are critical formulation variables affecting thermostability, while formulation concentration was not found to be as significant as the other variables. This result is corroborated by the statistical analysis results of the literature source (He et al., 2011c). Plots of pH values against presence of ions under different excipients are generated in Figure 1(d). Similar conclusions can be drawn from observations of Figures 1(c) and (d):

- pH value has the most effect on thermostability. High pH values (5.5 and 6.0) are more likely to have high hydrophobic exposure temperature and vice versa. When pH is 5.0, low *T*_h_ (<50 °C) is expected irrespective of the other factors which means the proteins are unstable. Effects of pH on mAb stability have been reported in previous work (Vermeer and Norde, 2000) and it could be explained by the low temperatures of unfolding that occur at low pHs (Vermeer et al., 2000).
- When pH is 5.5, the presence of Ca^2+^ can reduce the protein thermostability at high pH levels (5.5 and 6.0) under the condition without any excipient. However, when pH is 6.0, the presence of excipient, either proline or sucrose, will compensate the effect of Ca^2+^ and lead to high thermostability again. The same observations about the addition of ions decreasing protein thermostability and the effect of Ca^2+^ being more significant than the effect of Mg^2+^ have been reported in He et al. (2011c).

This section has shown that the CART tree method has correctly identified the main influences on mAb viscosity and thermostability, and therefore acts as a suitable input for the work on manufacturability indices, as discussed below.

## Manufacturability Index Results

In this work, three different multi‐criteria frameworks are proposed and implemented to derive manufacturability indices: “quantified overlay region”, “temporal operating window”, and “temporal multi‐criteria weighted score” framework. The manufacturability index is a set of numbers which can indicate the ease of manufacture during the final UF/DF step for each formulation candidate.

### Manufacturability Index Derived Using the Quantified Overlay Region Framework

#### Step 1: Generation of Stress Maps

**Figure 2:**
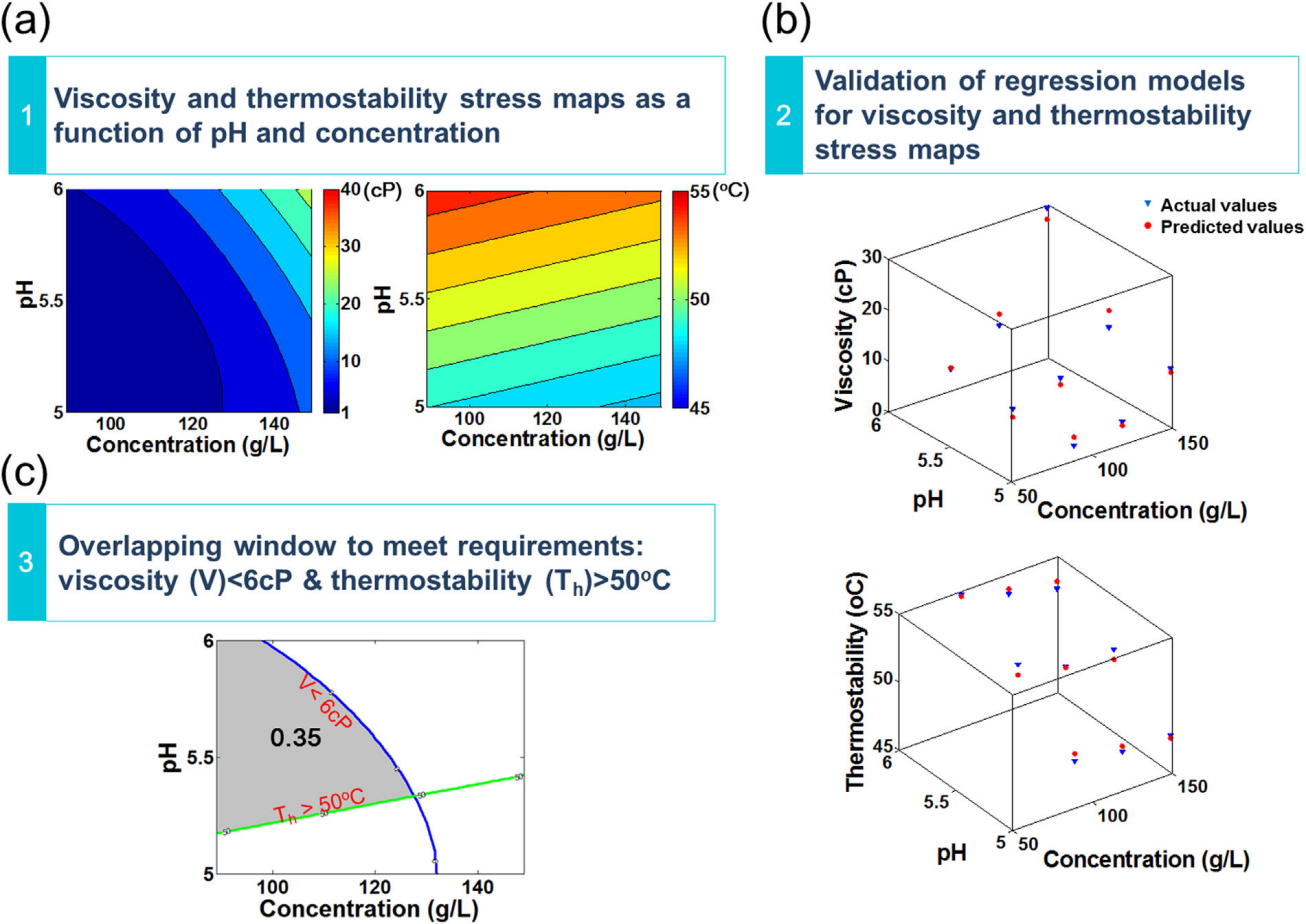
Methodology of quantified overlay region framework to generate manufacturability index for formulation of *no ion with no excipient*. (**a**) Generate viscosity and thermostability stress maps as functions of pH and protein concentration. (**b**) Validate the regression models for viscosity and thermostability stress map. Plots of actual values against the predicted values from regression models of viscosity and thermostability for formulation of *no ion with no excipient*. The blue triangles represent actual values while red dots represent predicted values. (**c**) Calculate manufacturability index for formulation of *no ion with no excipient* to meet both viscosity and thermostability requirements. The blue line is the contour line of viscosity of 6 cP, while the green line is the contour line of thermostability of 50 °C. The shaded area is the overlapping window to meet both requirements of viscosity <6 cP and thermostability >50 °C. The manufacturing index (0.35 in this example) is calculated as the ratio of the overlapping window area over the whole stress map area.

A stress map is a contour plot to explore how the performance or product quality (e.g., viscosity, thermostability in this work) changes with key process parameters (e.g., product concentration, pH). As described in the Section Methodology, there are two categorical variables: presence of ions and excipient. According to the combinations of ion and excipient, both viscosity and thermostability datasets were divided into nine sub‐datasets such as *no ion with no excipient* or *Ca^2+^ ion with sucrose excipient*. Nine polynomial models in the form shown in Equations (1) and (2) that describe viscosity and thermostability as a function of pH and protein concentration were built by polynomial regression for all formulation designs.

(1) $$V\left(x,y\right)=a_{00}+a_{10}x+a_{01}y+a_{11}xy+a_{20}x^{2}+a_{02}y^{2}$$

(2) $$T\left(x,y\right)=a_{00}+a_{10}x+a_{01}y$$

where *V*(*x,y*) represents the viscosity values, *T*(*x,y*) represents the thermostability values, *x* is protein concentration, and *y* is pH value. For the formulation of *no ion with no excipient*, the viscosity stress map and thermostability stress map are shown in Figure 2(a).

For each formulation design, the viscosity stress map indicates the prediction values of viscosity (cP) under different pH and protein concentrations while the thermostability stress map indicates the prediction values of hydrophobic exposure temperature (°C) under different pH and protein concentrations.

Both viscosity and thermostability stress maps will be used later to generate manufacturability indices for ranking formulation designs.

#### Step 2: Validation of Stress Maps

In order to validate the regression models for viscosity and thermostability stress map, plots of actual values against the predicted values from the regression models of viscosity and thermostability were generated and examined in terms of goodness of fit (*R* ^2^). Taking the formulation of *no ion with no excipient* as an example as shown in Figure 2(b), the triangles represent actual values while the dots represent predicted values. For the formulation of *no ion with no excipient*, the viscosity regression model has *R* ^2^ values of 0.95 indicating that approximately 95% of the variation in the response could be explained by the model while the thermostability regression model has *R* ^2^ values of 0.96. Similar goodness of fit values (91–97% for viscosity and 85–99% for thermostability) were observed for the eight other formulation condition combinations.

#### Step 3: Generation of Manufacturability Index by Quantifying the Overlay Region

Using stress maps, the predicted values for thermostability and viscosity at a particular pH and protein concentration for different formulation designs can be assessed. It is also possible to select the optimal pH and concentration range to meet specific limits for both thermostability and viscosity by overlapping the stress maps. If the critical values for viscosity and thermostability are 6 cP and 50 °C, respectively, Figure 2c shows the overlaid stress map for the formulation of *no ion with no excipient*. In Figure 2c, the curved (blue) contour indicates the critical viscosity value and the area below this contour is the desired area where viscosity values are lower than the critical value. The straight diagonal (green) contour indicates the critical *T*_h_ value and the area above this line is the desired area where *T*_h_ values are higher than critical value. The shaded area in Figure 2c, indicating the overlaid contour region, indicates the range of pH and concentrations that meet both thermostability and viscosity requirements for the formulation of *no ion with no excipient*. The manufacturability index based on the quantified overlay region framework is defined as the ratio of the overlay area to the whole stress map area. For example, for the formulation of *no ion with no excipient* highlighted in Figure 2c, the manufacturability index determined by the quantified overlay region framework is 0.35.

#### Selection of Optimal Formulation

**Figure 3:**
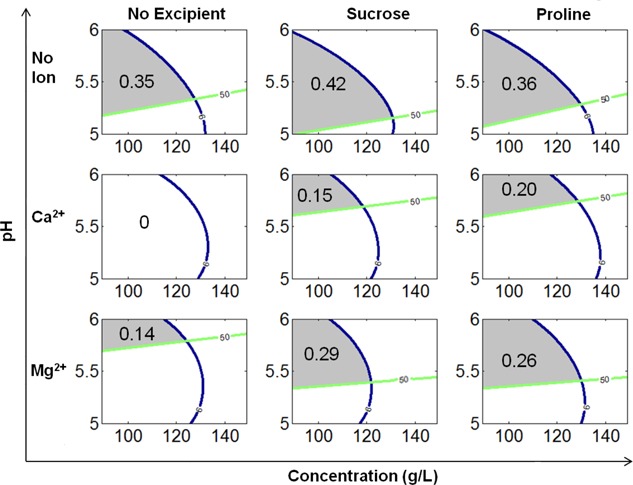
Manufacturability indices by quantified overlay region for all formulation conditions. The higher manufacturability index values indicate the higher tolerance capability to meet both requirements of viscosity <6 cP and thermostability >50 °C. The best formulation condition is no ion with sucrose, followed by *no ion with proline* and *no ion with no excipient*. For the formulation of *Ca^2+^ with no excipient*, the manufactuability index is 0 since there is no overlapping window for this formulation to meet both requirements.

The manufacturability indices generated using the quantified overlay region framework for all formulation conditions are shown in Figure 3. The indices were used to rank order the formulation designs, where a higher index value was considered more desirable given the wider allowable range of pH and concentration values to satisfy both thermostability and viscosity requirements. The best formulation condition was predicted to be *no ion with sucrose* followed by *no ion with proline* and *no ion with no excipient*. The worst formulation condition was found to be *Ca^2+^ ions with no excipient* with an index value of zero since there was no overlapping region of acceptable pH and protein concentration values meeting both thermostability and viscosity requirements. This framework is suitable for testing the robustness of formulation designs based on their overlay region size without consideration of UF/DF process parameters or a target final product concentration. Therefore, it can be used as the initial evaluation of formulation conditions without a specific target product profile.

Previous work has used overlay contour plots to explore the acceptable range of pH and salt concentration at particular limits for *T*_h_ and diffusion interaction parameter values (He et al., 2011a) and for *T*_h_ and viscosity values (He et al., 2011c). In this work, the analysis has been taken a step further by moving from visual inspection of the overlay regions to determining a numerical manufacturability index for each formulation combination based on the size of the overlay region. This provides a rapid methodology for ranking the different formulation conditions in a systematic manner.

### Manufacturability Index Derived Using the Temporal Operating Window Framework

#### Step 1: Operating Window Location Using the Thermostability Criterion

In this framework, the operating window is defined as the actual product concentration range and the optimal pH range experienced during the overconcentration and flush stages of the final UF/DF step. As described in the Section Problem Domain, the product stream after entering the UF/DF step is at 5 g/L, and during final UF/DF, it is concentrated to 150 g/L at the overconcentration step and diluted to 100 g/L at the flush step. In this work, only the upper values of the concentration range typically experienced during the overconcentration and flush stages (90–150 g/L) are considered since they have the potential to pose greater viscosity challenges during manufacture. The pH range is determined by the thermostability requirement for the final product specification of 100 g/L. Although thermostability temperatures will not be experienced during the final UF/DF step since the filtration operations are usually conducted at room temperature, the tolerance of hydrophobic exposure temperature (*T*_h_) may affect the protein product's long‐term stability during transition and storage. Therefore, it is meaningful to consider thermostability when choosing the best formulation conditions in the final UF/DF step as it is the last manufacturing step prior to fill‐finish operations.

**Figure 4:**
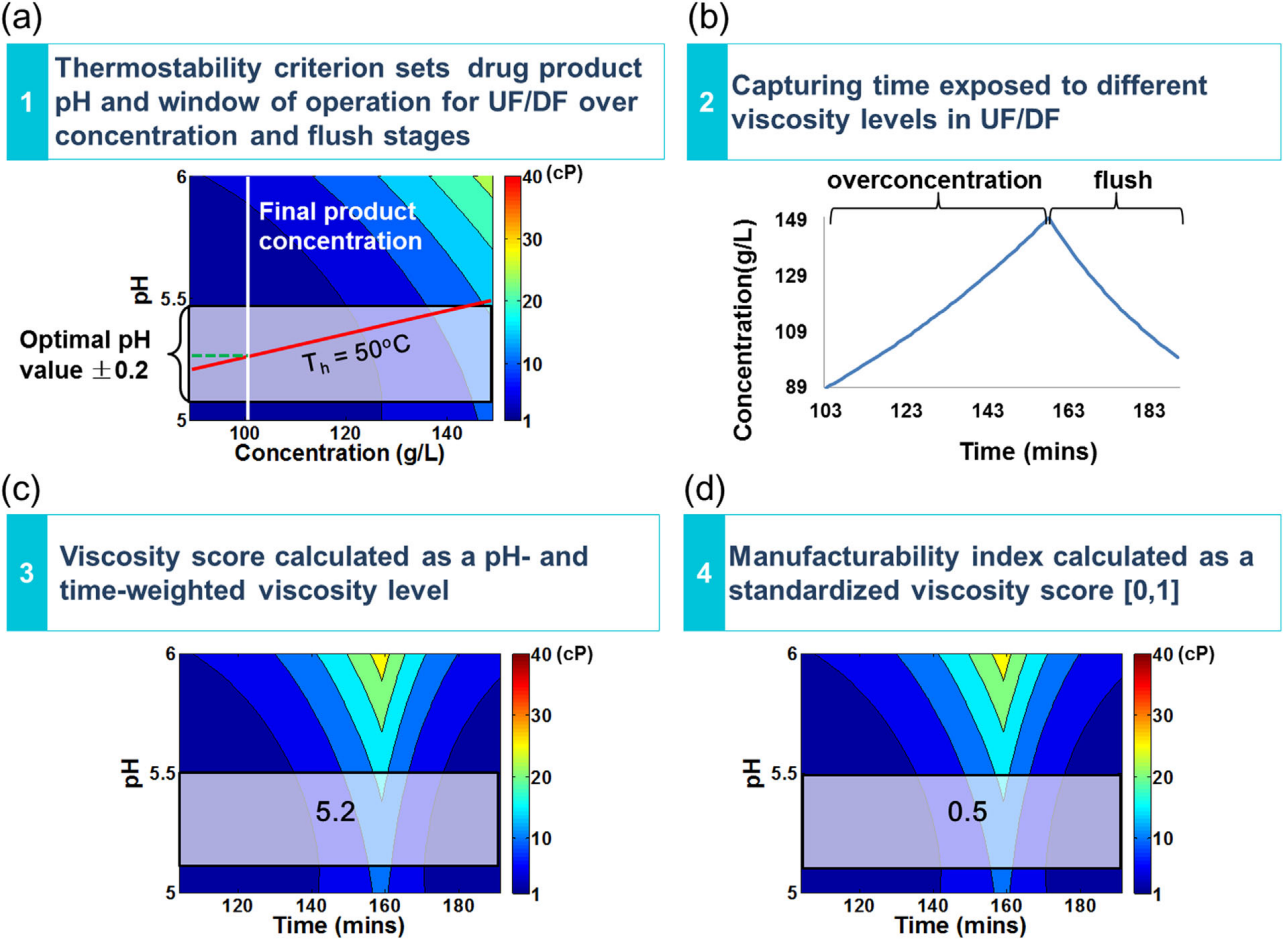
Methodology of temporal operating window framework to generate manufactuability index for formulation of *no ion with no excipient*. (**a**) Locate window of operation using the thermostability criterion. In the viscosity stress map for formulation of *no ion with no excipient*, the red line shows thermostability criterion *T*_h_ = 50 °C, while the white line shows the final product concentration of 100 g/L. The intersection of the red and white lines represents the pH for final product to meet *T*_h_ = 50 °C, which is 5.3. The operating range of pH is set to ±0.2, so the pH range of window of operation is 5.1–5.5. (**b**) Capture the time profile of product stream during overconcentration stage and flush stage in the final UF/DF step captured by the flux decay model. (**c**) Calculate the viscosity score as a pH and time weighted viscosity score. The viscosity score of formulation of *no ion with no excipient* is 5.2 cP. (**d**) Calculate manufacturability index as standard the viscosity scores into [0, 1]. The manufacturing index of formulation of *no ion with no excipient* is 0.5.

Since the thermostability criterion was set as *T*_h_ ≥50 °C as described in the Section CART Tree for Identification of Key Factors Influencing Viscosity, an operating window has been set up for each formulation design to identify the optimal pH range that meets the thermostability requirement. Figure 4(a) illustrates the identification of the operating window for the formulation condition of *no ion with no excipient*. In Figure 4(a), the vertical (white) contour indicates the final product concentration of 100 g/L and the diagonal (red) contour indicates the thermostability criterion. The intersection of the red and white contours indicates the pH value for the final product specification to meet the thermostability criterion. For example, for the formulation condition of *no ion with no excipient* illustrated in Figure 4a, the pH required is 5.3. Considering that a typical pH control range is within ±0.2 units, the operating window is determined by the ranges of concentration and pH that are 90–150 g/L and 5.1–5.5, respectively, as indicated by the rectangular overlay region in Figure 4(a).

#### Step 2: Processing Time Exposed to Different Viscosity Levels in Final UF/DF

In the final UF/DF step, the concentration of the product stream, and hence its viscosity, are dynamically changing over time. Furthermore, the flux through the membrane can be significantly lowered by membrane pore blockage and protein fouling that occurs as the concentration increases. In order to capture the information about the time exposed to different viscosity levels during UF/DF, the mathematical model for the filtrate flux proposed by Ho and Zydney (2000) was adapted and incorporated as Equation (3). In this work, the model has been adapted to not only capture the decreasing flux rate due to membrane fouling but also account for the impact of changing protein concentration over time on flux behavior:

(3) $$Q\left(t\right)=Q_{0}\left\{\exp\left(-\frac{\propto \Delta PC\left(t\right)}{\mu R_{m}}t\right)+\frac{R_{m}}{R_{m}+R_{p}}\left[1-exp\left(-\frac{\propto \Delta PC\left(t\right)}{\mu R_{m}}t\right)\right]\right\}$$

where $Q_{0}$is the initial volumetric flow rate through the clean membrane, μ is the solution viscosity, ΔP is the transmembrane pressure, $R_{m}$ is the clean membrane resistance, *t* is processing time, and∝ is a pore blockage parameter which is equal to the membrane area blocked per unit mass of protein convected to the membrane surface.

The resistance of the protein deposit over a particular region of the membrane $R_{p}$ is evaluated by Equation (4):

(4) $$R_{p}=\left(R_{m}+R_{p0}\right)\sqrt{1+\frac{2_{f}^{\prime }R^{\prime }\Delta PC_{b}}{\mu {\left(R_{m}+R_{p0}\right)}^{2}}t}-R_{m}$$

where $R_{p0}$ is the initial resistance of the protein deposit, $R^{\prime }$ is the specific protein layer resistance, and $f^{\prime }$ is the fraction of the proteins that contribute to the growth of the deposit.

The protein concentration during UF/DF, *C*(*t*) as a function of filtration flux and time is calculated by Equation (5):

(5) $$C\left(t\right)=\frac{C_{0}V_{0}}{V_{0}-\sum_{t=0}^{n}Q\left(t\right)\Delta t}$$

where $V_{0}$ is the initial protein stream volume, $C_{0}$ is the initial protein concentration, Δt is the time interval between *C*(*t*) and *C*(*t* *+ 1*).

The values of parameters for the flux decay model in this work are listed in Table III. These were derived from the literature (Ho and Zydney, 2000) and discussion with industry experts.

**Table III:** The parameters for flux decay model used in the work

Parameters	*Q_0_*	∝	ΔP	*C_0_*	*V_0_*	μ	*R_m_*	*f′R′*	*R_p0_*
Value	5000	0.33	5 × 10^5^	20	1050	0.001	7.5 × 10^11^	16 × 10^11^	3 × 10^10^
Unit	L/h	m^2^/kg	Pas	g/L	L	kg/m/s	m^−1^	m/kg	m^−1^

Using this model, the variation of protein concentration with processing time during the overconcentration and flush stages of the UF/DF step has been captured, as illustrated in Figure 4(b).

#### Step 3: Generation of Manufacturability Scores

With the model of processing time exposed to different viscosity levels in final UF/DF, the viscosity stress maps can be transformed as a function of pH and processing time. Figure 4(c) shows the transformed viscosity stress map for the formulation condition of *no ion with no excipient*. The operating window for the formulation condition of *no ion with no excipient* is determined by the ranges of processing time and pH which are 104–191 min and 5.1–5.5, respectively.

The average of viscosity values within the temporal operating window, termed the viscosity score, is calculated by Equation (6).

(6) $$V\text{iscosity}\;\text{score}=\frac{\sum_{\min\;pH}^{\max\;\text{pH}}\sum_{\min t}^{\max t}\mu \left(pH,\;t\right)}{N_{t}N_{pH}}$$

where μ(pH,t) is the viscosity regression model considering concentration‐time profile, which is a function of pH value and processing time *t*. *N_t_* and *N*_pH_ are the intervals of processing time *t* and pH, respectively, within the temporal operating window. The score indicates the average viscosity value of the product stream during the UF/DF step taking into account the operating range of pH and the time profiles of concentration. For example, for the formulation condition of *no ion with no excipient*, the viscosity score is 5.2 cP.

#### Step 4: Generation of Manufacturability Indices

The viscosity scores for all formulation conditions were standardized to [0, 1] so as to generate manufacturability indices using Equation (7).

(7) $$\text{Manufacturability index}=\frac{\text{viscosity score}-\text{worst score}}{\text{best score}-\text{worst score}}.$$

The reason for the transformation of viscosity scores is to obtain a set of uniform manufacturability indices to represent the ease of manufacture with the higher value the more desirable. The manufacturability index for the formulation condition of *no ion with no excipient* is 0.5.

#### Selection of Optimal Formulation

**Figure 5:**
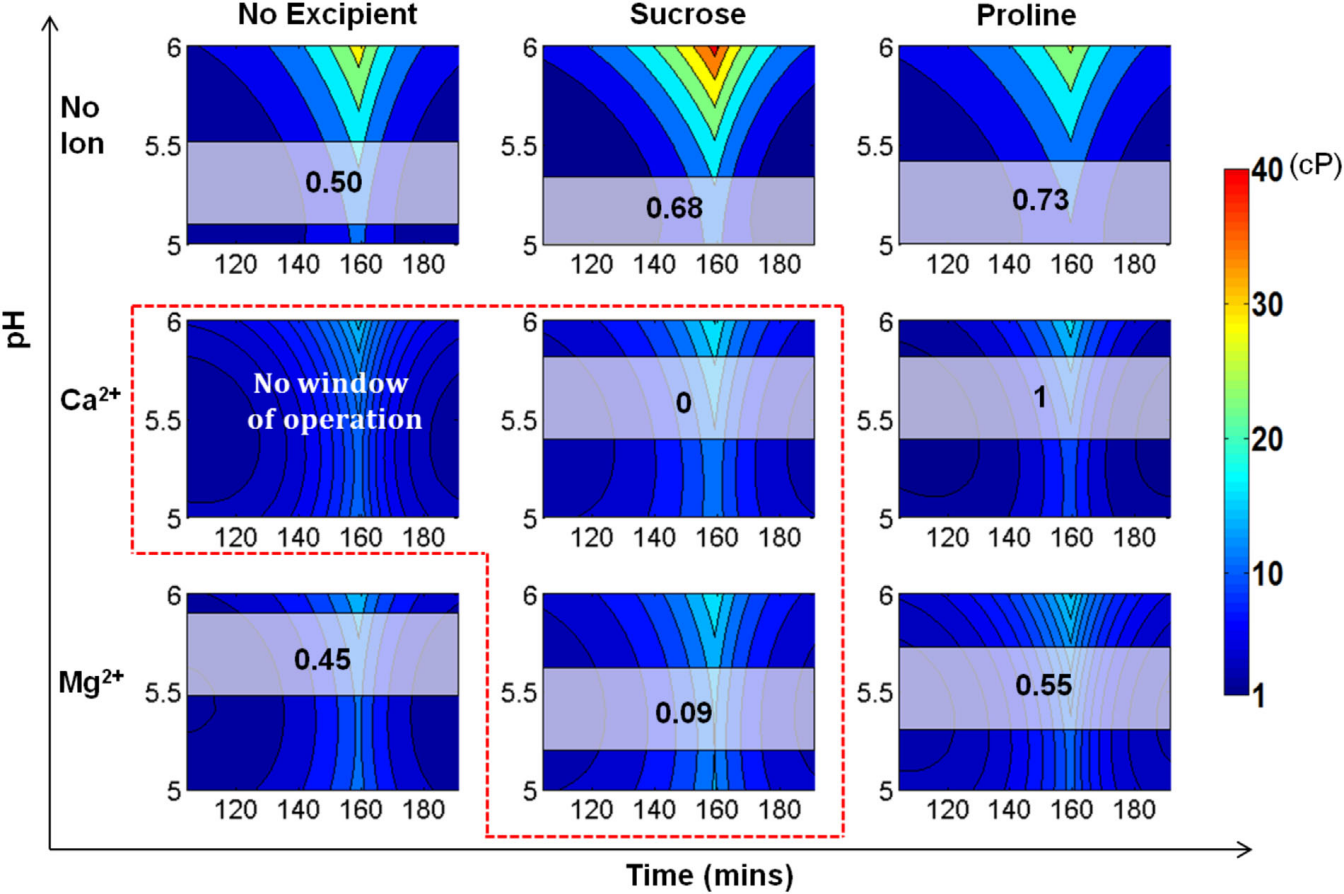
Manufacturability indices by temporal operating window for all formulation conditions. The index indicates the average viscosity value of the product stream during the UF/DF step taking into account the time profiles of pH and concentration. Each set of formulation conditions can be ranked according to their manufacturability indices where a higher index value indicates a more desirable outcome. The best formulation is *Ca^2+^ with proline*, followed by *no ion with proline* and *no ion with sucrose*. The three formulations in the red window are the worst three formulation conditions of all.

Manufacturability indices were generated for all formulation designs as shown in Figure 5. Each set of formulation conditions can be ranked according to their manufacturability indices where a higher index value indicates a more desirable outcome. From Figure 5, it can be seen that *Ca^2+^ ions with proline excipient* is the optimal formulation design with the highest manufacturability index, 1. Similarly, to the quantified overlay region results, there is no operating window for the formulation of *Ca^2+^ ions with no excipient* since the pH required for the final product to meet the thermostability criterion is 6.4 which is outside the experimental range of the dataset (5–6). Furthermore, if the critical acceptable viscosity value for the final UF/DF step is 6 cP (which translates to a manufacturability index of 0.14), three formulation designs would not be feasible: *Ca^2+^ ions with no excipient*, *Ca^2+^ ions with sucrose excipient*, and *Mg^2+^ ions with sucrose excipient*.

In this strategy, the rank order of formulation designs based on the temporal operating window framework is different to that from the quantified overlay region one. The reason for the difference is due to the additional consideration of the thermostability criterion. The position of the window is determined by the pH value for each formulation design to meet the thermostability criterion (50 °C). For example, the formulation of *no ion with sucrose* generally leads to the highest viscosity in all formulation designs. However, since sucrose can enhance the protein thermostability so that the pH value to meet thermostability criteria is the lowest, the formulation of *no ion with sucrose* is the second best design from the temporal operating window perspective.

Compared to the quantified overlay region framework in the Section Manufacturability Index Derived Using the Quantified Overlay Region Framework, this framework can rank and select formulation designs considering the process parameters (such as flux rate, membrane fouling, and overconcentration ratio) and time profiles during the final UF/DF step. This framework requires specification of the final product concentration.

### Manufacturability Index Derived Using the Temporal Multi‐Criteria Weighted Score Framework

In this section, thermostability was considered indicative of aggregation propensity where a high thermostability value and hence high‐protein conformational stability was taken as an indicator of low aggregation propensity (Goldberg et al., 2011).

#### Step 1: Standardizing Viscosity and Thermostability Values

**Figure 6:**
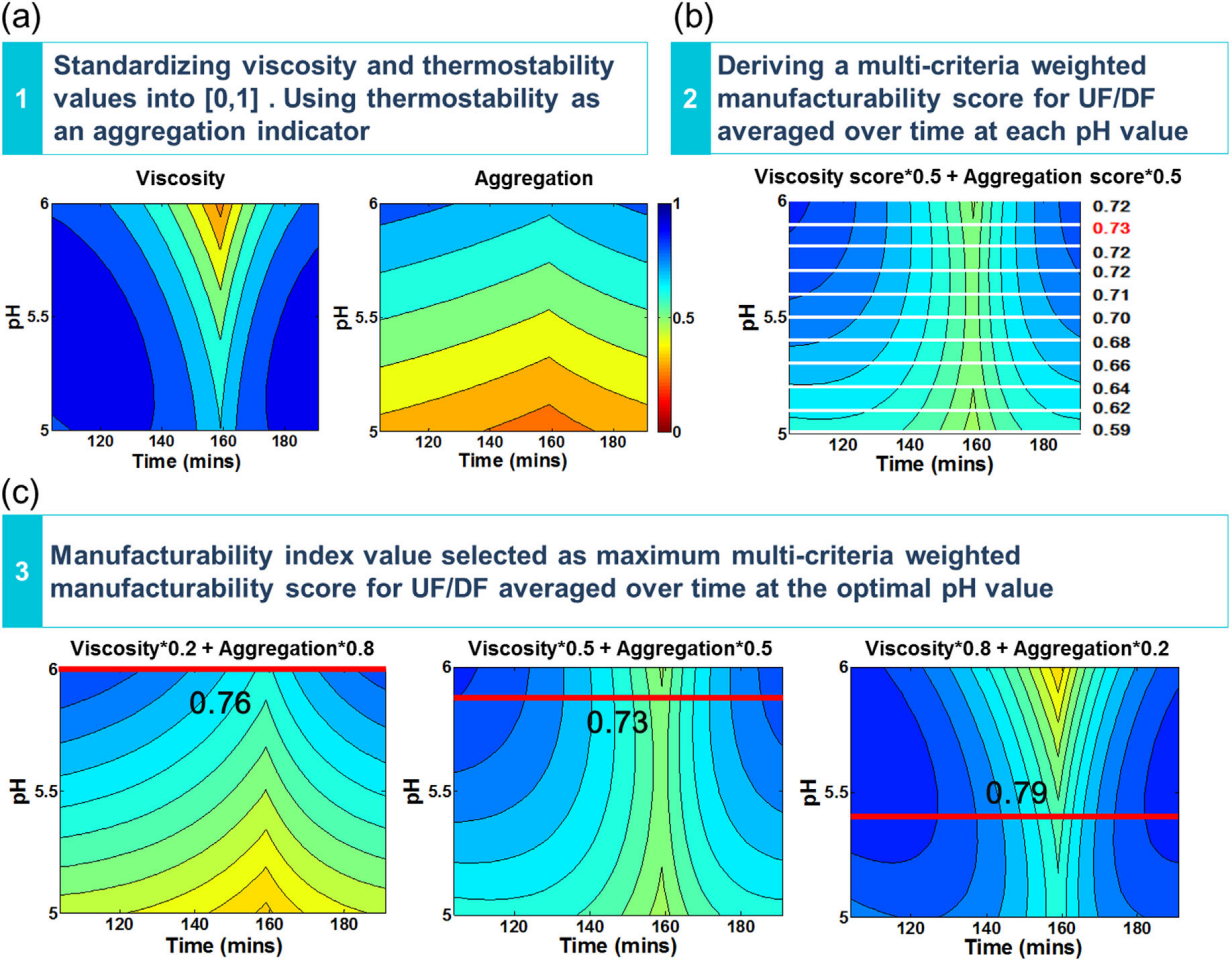
Methodology of temporal multi‐criteria weighted score framework to generate manufacturability index for formulation of *no ion with no excipient*. (**a**) Step 1: Standardize the real values of both viscosity and thermostability into [0, 1] to derive scores for viscosity and aggregation. Then, polynomial regression combined with the flux decay model was applied to the scores to generate stress maps for viscosity and aggregation as a function of time. In this framework, thermostability acts as an indicator of aggregation. (**b**) Step 2: Combine viscosity and aggregation scores using weighted sum method (WSM). Manufacturability, defined as weighted sum of viscosity and aggregation scores, is a function of pH and processing time. Different colors indicate different manufacturability levels. From blue to red, manufacturability is from easy to hard. Average manufacturability value for each pH calculated and the pH with the maximum average manufacturability value is the optimal pH value. When the weights for viscosity and aggregation are 0.5 and 0.5 separately, the optimal pH value for formulation *no ion with no excipient* is 5.9 and the maximum manufacturability is 0.73. (**c**) Step 3: Derive a multi‐criteria weighted manufacturability index for UF/DF averaged over time at the optimal pH value. The red lines indicate the optimal pH values for maximum manufacturability through the final UF/DF step. The optimal pH value changes with the weights. When *a* = 0.5, *b* = 0.5, the optimal pH is 5.9 and the maximum manufacturablity is 0.73; when *a* = 0.2, *b* = 0.8, the optimal pH is 6.0 and the maximum manufacturability is 0.76; when *a* = 0.8, *b* = 0.2, the optimal pH is 5.4 and the maximum manufacturability is 0.79.

Scores for viscosity and aggregation have been derived by standardizing the real values of viscosity and thermostability to [0, 1] using the same method as Equation (7) in order to eliminate the bias caused by different value ranges of aggregation and viscosity. If there are any extreme values or outliers in the viscosity and aggregation datasets, standard data processing methods should be used to remove outliers before standardizing. Then, similar to the Section Manufacturability Index Derived Using the Quantified Overlay Region Framework, stress maps that describe the scores for viscosity and aggregation as a function of pH and protein concentration were built by polynomial regression for all formulation designs. Using the same model of processing time exposed to different viscosity levels in the final UF/DF described in the Section Manufacturability Index Derived Using the Temporal Operating Window Framework, the stress maps for viscosity and aggregation can be transformed as a function of pH and processing time. Figure 6(a) shows the standardized viscosity and aggregation stress maps for the formulation condition of *no ion with no excipient*.

#### Step 2: Generate Multi‐Criteria Weighted Manufacturability Stress Maps and Scores

The weighted sum model, a popular multi‐criteria decision analysis method, was used to combine scores of aggregation and viscosity. Multi‐criteria manufacturability, defined as the weighted sum of viscosity and aggregation scores, is a function of pH and processing time. Figure 6(b) shows the manufacturability stress map for the formulation condition of *no ion with no excipient* when aggregation and viscosity are equally weighted. Different colors indicate different manufacturability levels. From blue to red, manufacturability is from easy to hard.

A set of temporal multi‐criteria weighted manufacturability scores were calculated by averaging the manufacturability scores over the UF/DF processing time for each pH value (Equation [(8)]). The manufacturability index value is selected as the maximum temporal multi‐criteira weighted score and hence the corresponding pH that maximized manufactruability is considered the optimal pH value.

(8) $$\text{Manufacturability score}=\overset{n}{\underset{t=i}{\sum }}(aV_{t}+bA_{t})\setminus \left(n-i\right)$$

where $V_{t}$ is viscosity score at time *t*, *A* is aggregation score at time *t*, *a* and *b* are weights assigned to the viscosity and aggregation scores respectively to reflect user priorities.

**Figure 7:**
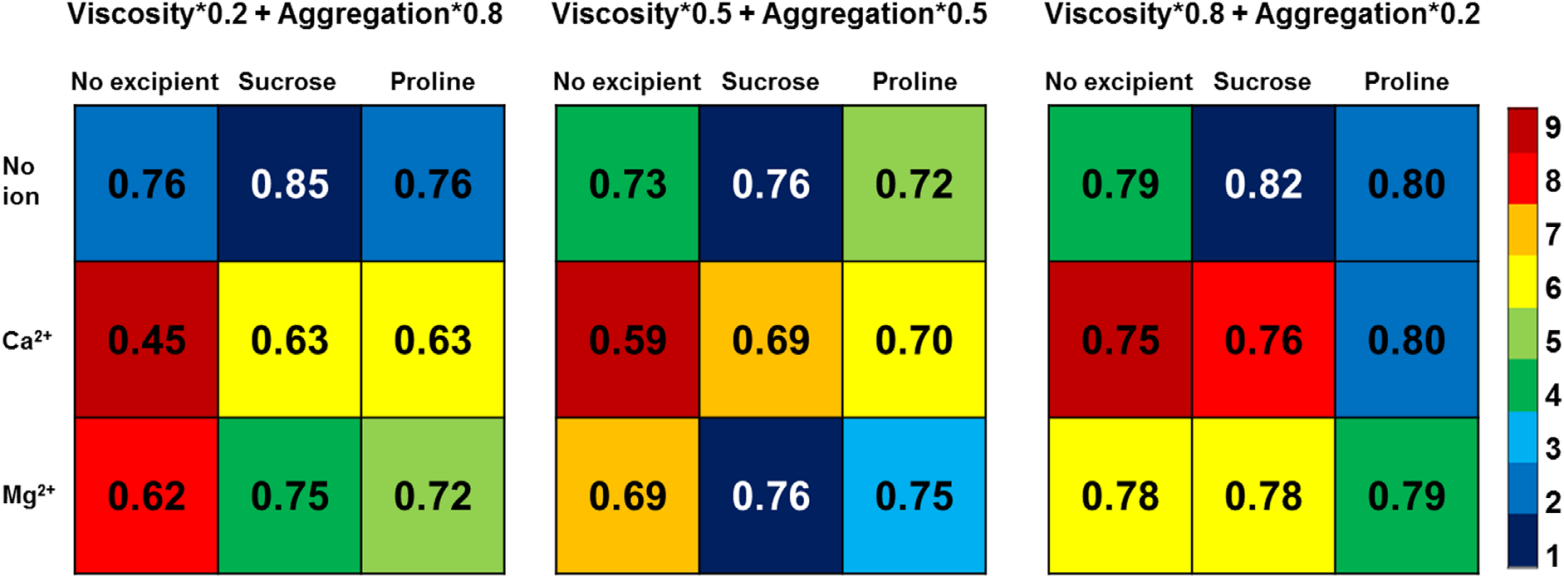
Manufacturability indices by temporal multi‐criteria weighted score framework for all formulation conditions. Comparison of all formulation conditions according to their manufacturability indices using multi‐criteria decisions with different weights on viscosity and aggregation. Each set of formulation conditions has been ranked when the weights on viscosity and aggregation are 0.2 and 0.8, 0.5 and 0.5, 0.8 and 0.2, respectively. Different colors indicate different ranking orders where 1 is the best formulation condition with maximum manufacturability index value and 9 is the worst.

#### Step 3: Select Manufacturability Index from Multi‐Criteria Weighted Manufacturability Scores

Figure 6(c) shows manufacturability stress maps by multi‐criteria decisions for the formulation condition of *no ion with no excipient*. The weights of viscosity and aggregation represent different user preference scenarios with different weights given to viscosity and aggregation: equally weighted (*a* = 0.5, *b* = 0.5), or viscosity considered more important than aggregation (*a* = 0.8, *b* = 0.2), or aggregation considered more important than viscosity (*a* = 0.2, *b* = 0.8). For example, when producing stable products, users may pay more attention to viscosity, therefore, in this case, the viscosity would have a higher weighting than aggregation. In contrast, when producing thermosensitive products, users may pay more attention to aggregation, therefore, the aggregation's weighting would be higher than viscosity. The red lines in Figure 6(c) indicate the optimal pH values that result in the maximum average manufacturability score for each of the weight scenarios. Comparing the three plots in Figure 6(c), it can be seen that the optimal pH value changes with the weights. When the weights of viscosity and aggregation are 0.2 and 0.8, respectively, the optimal pH is 6 and the maximum manufacturability is 0.76; when the weights are 0.5 and 0.5, the optimal pH is 5.9 and the maximum manufacturablity is 0.73; when the weights are 0.8 and 0.2, the optimal pH is 5.4 and the maximum manufacturability is 0.79. The trend suggests that when viscosity has a higher weighting, the optimal pH value is at the lower end of the range and conversely if aggregation has a higher weighting then the optial pH value is toward the upper end of the range.

#### Selection of Optimal Formulation

Figure 7 compares all formulation conditions according to their manufacturability indices using the multi‐criteria weighted score framework with different weights on viscosity and aggregation. Each set of formulation conditions has been ranked from 1 to 9 when the weights on viscosity and aggregation are 0.2 and 0.8, 0.5 and 0.5, 0.8 and 0.2, respectively. Different colors indicate different rank orders where 1 is the best formulation condition with the maximum manufacturability index value and 9 is the worst. Although the ranking of formulation conditions is changing with the weightings, several generic conclusions can be drawn:

- For all the three sets of weights on viscosity and aggregation, the best formulation condition is always *no ion with sucrose*. This can be attributed to the fact that sucrose improves the aggregation score dramatically whilst only lowering viscosity score slightly. As a result, the maximum weighted sum of viscosity and aggregation is always in the formulation condition of *no ion with sucrose*.
- The worst formulation condition in all three weighting scenarios is always *Ca^2+^ with no excipient*. Although Ca^2+^ can benefit the viscosity score, its negative effect on aggregation is much more significant so that the lowest weighted sum of viscosity and aggregation is always in this formulation.
- When the weights of viscosity and aggregation are 0.2 and 0.8, the best formulation condition is *no ion with sucrose* followed by the joint ranking of *no ion with no excipient* and *no ion with proline*. The worst formulation condition is *Ca^2+^ with no excipient*, followed by *Mg^2+^ with no excipient* and then both *Ca^2+^ with sucrose* and *Ca^2+^ with proline*. This result reveals that when the weight of aggregation is higher than viscosity, the optimal formulation conditions favor no ions or the presence of excipient. This result also reinforces previously reported observations that excipients can increase thermostability whilst ions can decrease thermostability (He et al., 2010b). This result is consistent with the quantified overlay region result in the Section Manufacturability Index Derived Using the Quantified Overlay Region Framework.
- When the weights of viscosity and thermostability are 0.8 and 0.2, the best formulation condition is *no ion with sucrose* followed by the equally ranked *no ion with proline* and *Ca^2+^ with proline*. The worst condition is *Ca^2+^ with no excipient*, followed by *Ca^2+^ with sucrose*, *Mg^2+^ with no excipient* and *Mg^2+^ with sucrose*. This result reveals that when the weight of viscosity is higher, the optimal formulation conditions favour the presence of proline. This observation is corroborated by studies indicating that proline effectively inhibits protein aggregation due to the formation of an ordered, amphipathic supramolecular assembly (Kumar et al., 1998). This result is consistent with the temporal operating window result in the Section Manufacturability Index Derived Using the Temporal Operating Window Framework.
- When viscosity and aggregation are equally weighted, the formulation conditions of *Mg^2+^ with sucrose* and *no ion with sucrose* are tied in terms of attractiveness followed by *Mg^2+^ with proline*. The worst formulation condition is *Ca^2+^ with no excipient*, followed by *Mg^2+^ with no excipient* and *Ca^2+^ with sucrose* equally. This result reveals that when the weights of viscosity and aggregation are equal, there will be more complicated trade‐offs between the presence of ions and excipients so that the optimal formulation conditions favour the presence of *Mg^2+^* with excipients.

## Conclusion

It is important to be able to select candidates early on based on both the ideal formulation for clinical efficacy and uptake but also for manufacturability. This work presents the concept of manufacturability indices as early predictors to select the optimal formulation designs for selected candidates. Three quantitative index‐generation frameworks (quantified overlay region, temporal operating window, and temporal multi‐criteria weighted score) have been proposed and demonstrated in this work. The three frameworks analyzed the same DoE datasets from static, temporal, and weighted multi‐criteria perspectives, respectively, to generate manufacturability indices. Each framework could rank the formulation designs and choose the optimal formulation of buffer conditions with the best potential manufacturability for the final UF/DF step in high‐concentrated mAb manufacturing processes. Although the rank order is different, all three frameworks have their own advantages and applications.

The advantage of the quantified overlay region framework is its simplicity and directness. Since this framework does not relate to specific unit operation steps and final product concentration it can be used as the initial evaluation of formulation conditions. The advantage of the temporal operating window framework is that it can be used as an advanced predictor for a particular product and specific UF/DF processing parameters. The advantage of the multi‐criteria decision framework is its flexibility. The weightings of viscosity and stability are adjustable so as to satisfy different manufacturing requirements and user priorities. This framework also considers the process constraints, time profiles during the final UF/DF step and the final product concentration. This framework can be extended to account for optimal manufacturing for multiproduct facilities.

Another contribution of this work is introducing the CART decision tree method to explore the influence of different formulation conditions on the solution viscosity and thermostability and to identify the critical factors for both properties. The results of decision tree analysis are meaningful for future high‐throughput experiment design and formulation improvement.

This work has laid the foundation for quantitative methodologies to derive manufacturability indices from biophysical datasets. It has demonstrated the utility of these indices for formulation screening studies and their capability to rank order formulation conditions considering viscosity and thermostability or aggregation propensity whilst identifying problematic formulations early on. The development of high throughput assays for measuring such attributes may also mean that similar stress datasets could in future be created for multiple candidates prior to entering the development cycle. Hence the frameworks proposed in this paper could in future be used during molecular/developability assessments (Lorenz et al., 2014, Sharma and Kelley, 2015) carried out during the molecule discovery phase and contribute to candidate selection as well as formulation design. The measurements of manufacturability are of course not restricted to viscosity and thermostability; they could include other biophysical properties such as hydrophobicity and solubility, quality attributes such as degradation and charge variant levels as well as productivity measurements. Future work will examine how best to integrate these additional datasets into an overall manufacturability index that can be considered during developability assessments so as to help de‐risk, streamline and accelerate biopharmaceutical development.

Funding from the UK Engineering & Physical Sciences Research Council (EPSRC) for the EPSRC Centre for Innovative Manufacturing in Emergent Macromolecular Therapies is gratefully acknowledged. Financial support from the consortium of industrial and governmental users is also acknowledged.
